# Vocal copying of individually distinctive signature whistles in bottlenose dolphins

**DOI:** 10.1098/rspb.2013.0053

**Published:** 2013-04-22

**Authors:** Stephanie L. King, Laela S. Sayigh, Randall S. Wells, Wendi Fellner, Vincent M. Janik

**Affiliations:** 1 Sea Mammal Research Unit, School of Biology, University of St Andrews, St Andrews, Fife KY16 8LB, UK; 2 Biology Department, Woods Hole Oceanographic Institution, Woods Hole, MA 02543, USA; 3 Chicago Zoological Society, c/o Mote Marine Laboratory, 1600 Ken Thompson Parkway, Sarasota, FL 34236, USA; 4 The Seas, Epcot, Walt Disney World Resort, 2016 Avenue of the Stars, EC Trl. W-251, Lake Buena Vista, FL 32830, USA

**Keywords:** vocal learning, *Tursiops*, imitation, communication

## Abstract

Vocal learning is relatively common in birds but less so in mammals. Sexual selection and individual or group recognition have been identified as major forces in its evolution. While important in the development of vocal displays, vocal learning also allows signal copying in social interactions. Such copying can function in addressing or labelling selected conspecifics. Most examples of addressing in non-humans come from bird song, where matching occurs in an aggressive context. However, in other animals, addressing with learned signals is very much an affiliative signal. We studied the function of vocal copying in a mammal that shows vocal learning as well as complex cognitive and social behaviour, the bottlenose dolphin (*Tursiops truncatus*). Copying occurred almost exclusively between close associates such as mother–calf pairs and male alliances during separation and was not followed by aggression. All copies were clearly recognizable as such because copiers consistently modified some acoustic parameters of a signal when copying it. We found no evidence for the use of copying in aggression or deception. This use of vocal copying is similar to its use in human language, where the maintenance of social bonds appears to be more important than the immediate defence of resources.

## 1. Introduction

Vocal production learning enables animals to copy novel sounds in their environment or to develop their own distinctive calls, avoiding overlap with those heard before [1]. Most commonly, vocal learning leads to convergence in sound parameters between individuals. A good example of this can be found in bird song dialects [2] or in the development of group-specific contact calls [3–7]. The exchange of such shared calls between individuals can be aggressive or affiliative in nature. While contact calls are known to be affiliative [7], song type matching in song birds tends to have an aggressive connotation [8]. Song sparrows, for example, use song type matching when defending their territory against an unknown male, but avoid it when interacting with known neighbours with whom they use more subtle repertoire matching [9,10]. Repertoire matching, i.e. the use of a shared song type while avoiding a reply with the same song type, may allow the addressing of a neighbour in a more affiliative or neutral way.

In most instances, these interactions occur with calls that are shared by more than one individual. In the case of contact calls, the common call belongs either to a group or a pair of animals. In bird song, animals have individual repertoires where each song type is shared with other individuals, but the overall composition of the repertoire may be unique. Production rates for each shared call or song type are usually similar across the individuals that share it. Individual call or song types survive in populations as cultural traditions that can outlive the animals that produce them at any one time [11].

The signature whistle of the bottlenose dolphin stands out from these examples in that it seems to be more individually specific. Bottlenose dolphins produce a large variety of narrow-band frequency-modulated whistles and pulsed sounds for communication [12]. As part of their repertoire, each individual also develops an individually distinctive signature whistle [13,14] that develops under the influence of vocal learning [15–17]. Individuals listen to their acoustic environment early in life and then develop their own novel frequency modulation pattern or contour for their signature whistle [15]. The result is a novel and unique modulation pattern that identifies the individual even in the absence of general voice cues [18]. Interindividual variation in signature whistles is much larger than that found in recognition signals of other species [19].

Bottlenose dolphins live in fluid fission–fusion societies with animals forming a variety of different social relationships [20]. This social organization, coupled with restrictions in underwater vision and olfaction, has led to natural selection favouring designed individual signature whistles [12,14] instead of relying on the by-product distinctiveness of voice features [19]. The signature whistle tends to be the most commonly used whistle in each individual's repertoire accounting for around 50 per cent of all whistles produced by animals in the wild [21]. Bottlenose dolphins are, however, able to learn new sounds throughout their lives [22], and conspecifics occasionally imitate the signature whistles of others [23]. Thus, one animal's signature whistle can form a minor part of another animal's vocal repertoire as a result of copying [17,23,24]. Signature whistle copying is, however, rare [23,25–27], albeit significantly more common than expected by chance [25]. As such, each signature whistle forms only a major part of one animal's repertoire, allowing it to be a label for that particular individual when copied.

Nevertheless, the function of copying events remains unclear. It has been argued that copying of signature whistle types is equivalent to addressing other individuals. Such addressing can be affiliative or aggressive. Unlike songbirds, delphinids are not territorial and do not sing. Instead, they use their acoustic signals in the context of social interactions and group cohesion [12]. Bottlenose dolphins have low rates of aggression towards close associates and higher ones towards social competitors, for example among male alliances [20]. Investigating who is copying who can therefore give us information on the signal value of copying. In addition to affiliative and aggressive functions, a third hypothesis for whistle copying is that it is used as a deceptive form of signalling [28]. For example, deceptive signature whistle copying by male dolphins could allow them to gain access to females guarded by other males or to avoid directed aggression from a male alliance [29]. It appears that copies are sufficiently rare to allow for such a use without jeopardizing the reliability of signature whistles as identity signals.

To investigate these three hypotheses, the occurrence of signature whistle copying was studied in captive and briefly captured and subsequently released wild bottlenose dolphins. We hypothesized that if signature whistle copying is affiliative it should only occur between close associates. Alternatively, copying in an aggressive context should be more common between animals that are less closely associated. Furthermore, copies used in a deceptive way should ideally not be recognizable as copies, whereas in affiliative or aggressive contexts, they could be recognizable as such. We also investigated the temporal aspects of whistle copying given the importance of signal type matching in other species.

## 2. Material and methods

### (a) Social and acoustic data from the wild

Data were collected from wild bottlenose dolphins around Sarasota Bay, FL, USA between 1984 and 2009. The amount of time animals are sighted together can be used to give a measure of their association. The half-weight ratio coefficients of association (CoA) [30] is defined as CoA = 2*N_ab_*/*N_a_* + *N_b_*_,_ in which *N_ab_* is the number of times individuals A and B have been seen together, *N_a_* is the number of times individual A has been seen without B, and *N_b_* is the number of times individual B has been seen without A. CoAs were calculated for all study animals from data gained during regular, systematic photographic identification surveys of dolphins. CoAs given for each pair of animals caught together are from the year the recordings were taken. Wild bottlenose dolphin acoustic recordings were collected during capture–release events for health assessments and life-history studies in Sarasota Bay [31]. One such event takes on average 108 min from the time the net is set to the time the individual is released. During these events, animals were physically restrained and frequently out of visual sight, but not acoustic range, of one another. The signature whistle of an individual is the most common whistle type emitted in such isolation conditions [14]. The Sarasota Dolphin Research Programme has now accumulated a catalogue of whistles from over 250 individual dolphins from the resident community in Sarasota Bay since 1975 [14], many of which were recorded in multiple capture–release sessions. We compared all whistles produced by an individual with the signature whistles of all others in the same capture set in order to identify copying events. Ages of animals were known from long-term observations [32] or from analysing growth rings in teeth [33].

The vocalizations of each individual were recorded via a suction cup hydrophone, allowing the identification of the caller for each recorded call. Either custom-built or SSQ94 hydrophones were used (High Tech Inc.). Between 1984 and 2004, the acoustic recordings were taken with either Marantz PMD-430 or Sony TC-D5M stereo-cassette recorders (frequency response of recording system: 0.02–18 kHz ± 5 dB) or Panasonic AG-6400 or AG-7400 video-cassette recorders (frequency response of recording system: 0.02–25 kHz ± 3 dB). For recordings taken from 2005 onwards, a Sound Devices 744T digital recorder was used (sampled at 96 kHz, 24-bit, frequency response of recording system: 0.02–48 kHz ± 1 dB).

The first step of analysis consisted of visual comparisons of spectrograms of 205 h and 23 min of acoustic recordings of temporarily caught and released, wild bottlenose dolphins by one observer in order to identify copying events within each capture set. The total recording time inspected in this way was 110 h and 55 min for pairs of animals caught together with low association levels (CoA < 0.5) and 94 h and 28 min for pairs of animals caught together with high association levels (CoA > 0.5). The second step involved a detailed analysis of 32 h and 12 min (table 1) of recordings where vocal copying had been found. These contained a total of 10 219 whistles, which is the dataset on which this in-depth analysis is based.

**Table 1.** Pairs of animals involved in signature whistle copying events, with the animal producing copies in bold. The mean similarity values are given for each animal's signature whistle when compared with the vocal copy. The copier's own signature whistles had low similarity scores with the copy while the signature whistles of the copied animals had high similarity scores with the copies (see the electronic supplementary material, figure S1).

pair	sex	relationship	CoA	age	recording time (min)	no. of vocal copies	average similarity values
**1. Calvin Ranier**	MM	associates	1^a^	1528	7070	13—	1.54.5
**2a. FB26** **2b. FB48**	MM	alliance partners	0.8	3129	93101	385	1.0/3.2^b^1.0/3.5^b^
**3. FB114** FB20	MM	associates	0.07	1615	5195	4—	2.43.3
**4. FB90**FB122	FM	mothercalf	0.98	254	9292	17—	1.33.3
**5. FB65**FB67	FF	calfmother	0.67	621	7070	1—	1.23.6
**6. FB228**FB65	MF	calfmother	0.95	521	106106	8—	1.13.5
**7. FB5**FB55	FF	mothercalf	1.0	293	8585	17—	1.33.3
**8a. FB358b. FB93**	FF	mothercalf	0.9	323	9292	24	1.7/3.7^b^2.5/3.2^b^
**9. FB71**FB95	FF	mothercalf	1.0	281	9797	13—	1.03.3
**10. FB5**FB155	FF	mothercalf	0.56	292	7979	40—	1.03.5
**11. FB9**FB177	FF	mothercalf	0.9	20	105105	9—	1.23.4

^a^These animals were permanent residents in a captive facility.

^b^Where both animals copied one another the average similarity value for that animal's own signature with the copy it produced of the other animal's signature whistle is given first (low number) followed by the average similarity value for that animal's own signature whistle with the copy produced by the other animal in the pair (larger number).

### (b) Social and acoustic data from captivity

To investigate the social context of copying, four captive adult males were recorded at The Seas Aquarium, Lake Buena Vista, FL, USA, during May–June 2009. One male, Ranier, was estimated to be 28 years old and was collected at approximately 3 years of age in the northern Gulf of Mexico. The other males were Calvin (15 years old), Khyber (18 years old) and Malabar (8 years old), who were all captive born. All four animals had been together for 3.5 years at the start of the study; Ranier and Calvin had been together for 6 years. Vocalizations of these dolphins were recorded with two HTI-96 MIN hydrophones (frequency response: 0.002–30 kHz±1 dB) and two CRT hydrophones (C54 series; frequency response: 0.016–44 kHz±3 dB) onto a Toshiba Satellite Pro laptop using a four channel Avisoft v. 416 UltrasoundGate recording device (sampled at 50 kHz, 8 bit).

A total recording time of 16 h for the four males was analysed. The length of recording time when copying between pairs could be identified (as determined by their positions in the pool system) was as follows: 16 h (100%) for Ranier and Calvin, Ranier and Malabar, Khyber and Calvin and Khyber and Malabar; 14 h (87.3%) for Ranier and Khyber and 2 h (12.7%) for Calvin and Malabar. The caller was identified, using passive acoustic localization [23]. The social association of male pairs at The Seas was evaluated by measuring synchrony in their swimming patterns [34]. A focal animal instantaneous sampling method was used with an observation period of 7.5 min and a 15 s interval. At each 15 s interval, the focal animal's synchrony status was assessed relative to each other animal in the group. Observations took place 5 days per week between 08.00 and 18.00, and each animal served as the focal animal once each day in an order determined by a balanced, randomly ordered schedule. Observations were made between January 2009 and June 2009 when all four dolphins were together in the same pool.

### (c) Identifying copying events

Initially, one observer (S.L.K.) compared all whistles in a given captured or captive group with each other, and identified all occurrences where the same whistle type was being produced by more than one animal by inspecting spectrograms (fast Fourier transform (FFT) length 512, overlap 100%, Blackmann–Harris window) in Adobe Audition v. 2.0 (Adobe Systems). Five naive human observers, blind to context and animal identity, were then used to rate the similarity of each copy of a signature whistle to the original signature whistle (the whistle as produced by its owner) and to the copier's own signature whistle. Visual classification was used as it is more reliable than computer-based methods in dolphin whistle classification [14,35] and is frequently used in animal communication studies [2,36]. The five observers were given the extracted contours (frequency modulation pattern) of the whistles as plots of frequency versus time and were asked to rate whistle similarity using a five-point similarity index ranging from 1 (dissimilar) to 5 (similar). Only copied whistles that reached a mean similarity score of more than 3 with the original signature whistle and less than 3 with the copier's own signature whistle were deemed copies and included in the analysis. A value of 3 indicates a relatively high similarity as indicated in previous studies [25,29,37].

### (d) Acoustic analysis

The whistle contours of every copy as well as of randomly chosen exemplars of signature whistles of both interacting individuals were extracted using a supervised contour extraction programme [38], with a time resolution of 5 ms. From the contours, the following parameters were measured: start frequency, end frequency, minimum frequency, maximum frequency, frequency range, duration and mean frequency. One further parameter, number of loops, was read directly from the spectrogram where applicable. A loop was defined as a repeated modulation pattern within a signature whistle that could be separated by periods of stereotyped, discrete segments of silence. These periods of silence were taken to be 250 ms or less, which is the maximum inter-loop interval found in this population [39].

### (e) Statistical analysis

All statistical procedures were conducted in R (R project for statistical computing; GNU project). Acoustic parameters were analysed by first testing for normality using the Lilliefors (Kolmogorov–Smirnov) test. Depending upon the outcome, either the Mann–Whitney test or a Welch's *t*-test was used to compare differences between parameters of the copies with the original signature whistles and the copier's own signature whistle. A sampling statistic was then created by multiplying these test statistics together, which created a combined test statistic for all parameters. This allowed comparisons of overall difference between two whistle types. A permutation test was used to shuffle the acoustic parameter measurements of the copies with those of the original signature whistles within each pair of animals. This was carried out to test whether the combined acoustic parameter statistic was significantly different from a random distribution. Ten thousand permutations were performed to calculate the distribution of the test statistic under the null hypothesis (random distribution), and the observed test statistic was then compared with this random distribution. A two-tailed test was used with a Bonferroni-adjusted significance level of *p* < 0.002. In addition, all parameters were used in a non-metric multi-dimensional scaling analysis with a good STRESS fit of 0.04.

A permutation test was also used to test whether signal copying only occurred between affiliated pairs of animals. This involved shuffling the CoAs of the pairs of animals who produced vocal copies (*n* = 11) with those that did not (*n* = 191). Many of the individuals who copied were also in pairs with other animals where copying was not present. The sampling statistic of interest was the mean CoA for the pairs involved in signal copying. Ten thousand permutations were performed to calculate the distribution of the test statistic under the null hypothesis that the CoAs of copiers were randomly distributed. The observed test statistic was then compared with the random distribution.

Permutation tests were also performed on the timing of copies after the original signature whistle. The times of copies (*n* = 108) were shuffled with the times of the copier's own signature whistles given in response to the copied signature whistles (*n* = 1651). The random distribution was calculated from 10 000 permutations under the null hypothesis that there was no difference between the timing of copies of signature whistles after the occurrence of the template whistle and the timing of the copier's own signature whistle after the occurrence of the template whistle. The observed test statistic (mean time between original signature whistle and copy) was compared with the random distribution.

## 3. Results

### (a) Who copies whom?

In total, 85 different capture–release events of wild dolphins were analysed, comprising 121 individuals in different group compositions. Of these individuals, 48 were sampled on more than one occasion (range: 2–7). Of the 85 capture–release events analysed, 11 consisted of single male–male pairs, 31 consisted of single mother–calf pairs and the remaining 43 consisted of groups of different compositions. These compositions included two or more adults of the same or both sexes, mother–calf pairs with other adults and groups of mother–calf pairs.

**Figure 1.**
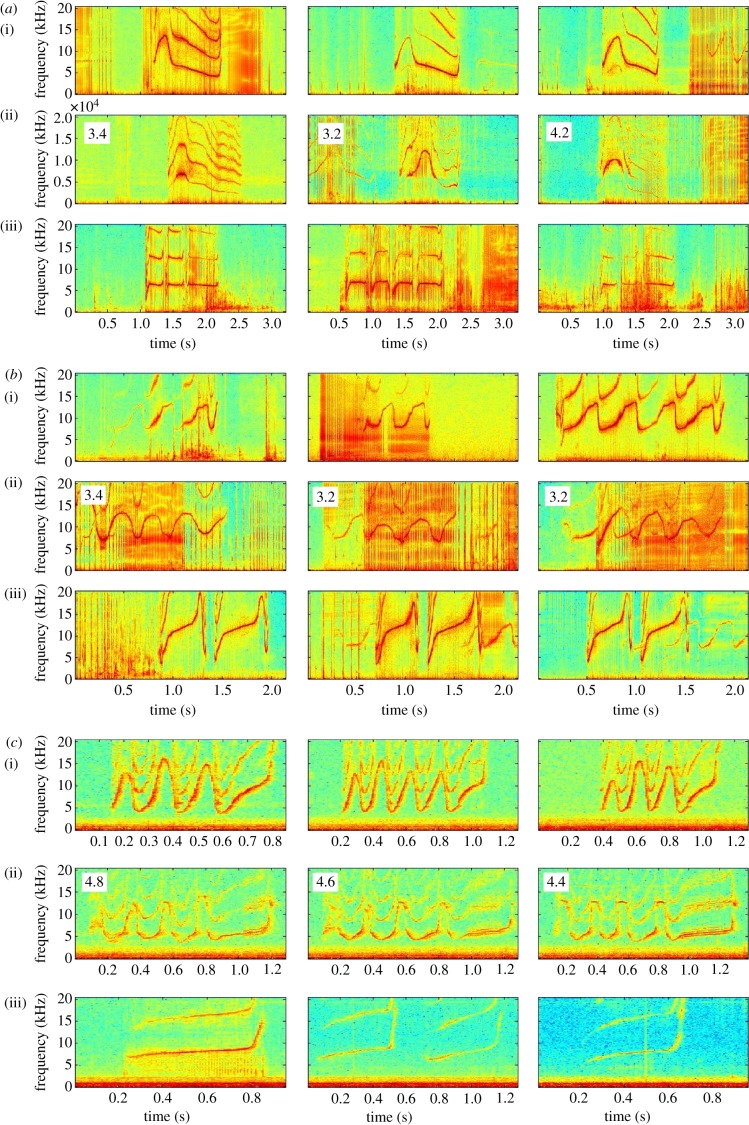
Spectrograms showing three examples each of the (i) signature whistle of the animal being copied, (ii) signature whistle copies and (iii) the signature whistle of the copier; sampling rate: 40 000 H*z*, FFT length: 1024, Hanning window function. Numbers on the middle spectrograms give the mean human observer similarity scores between the original and the copy for each pair of whistles on a scale from 1 (not similar) to 5 (very similar). (*a*) Vocal interaction of a mother–calf pair. The mother, FB65, was the signature whistle owner (i) and the male calf, FB228, was the copier (iii). The male produced copies are in row *a* ii. (*b*) Vocal interaction of another mother–calf pair. The male calf, FB122, was the signature whistle owner (i) and the mother, FB90, was the copier (iii). The copies she produced are in row *b* ii. (*c*) Vocal interaction of a male–male pair from The Seas. The first adult male, Ranier, was the signature whistle owner (i) and the second adult male, Calvin, was the copier (iii). The copies he produced are in row *c* ii. (Online version in colour.)

As in previous studies [14,40], each bottlenose dolphin almost exclusively used its own, individually distinctive signature whistle during capture–release events. Whistle rates were generally high at these events, with a mean of 5.3 whistles per minute per individual. In 10 of 85 different capture–release sets, however, individuals were found occasionally copying the signature whistle of another animal in the set (mean rate in sets with copying: 0.18 copies per minute per individual). This occurred in 10 of 179 pairs of animals recorded from 1988 through 2004, consisting of two of the 11 male–male pairings and eight of the 31 mother–calf pairs. In some instances, both members of a pair copied one another (figure 1 and table 1; electronic supplementary material, figure S1). The total number of individuals who produced vocal copies was therefore 12. The five human judges who viewed frequency contour plots to quantify similarity of the copies with both the originals and the copier's own signature whistles showed statistically significant agreement (*κ* = 0.42, *z* = 29.9, *p* < 0.0001) [41]. Similarity values for all copies are given in table 1.

**Figure 2.**
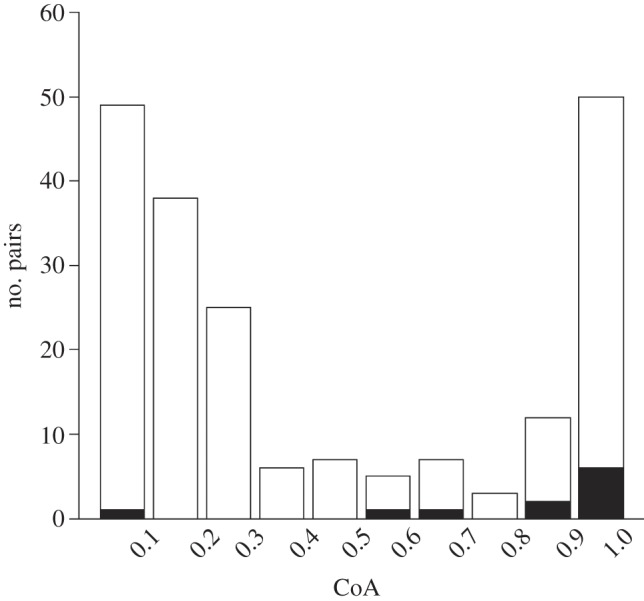
Coefficients of associations (CoA) of the pairs of animals that copied (black) and did not copy (white). The *y*-axis is the number of pairs of animals (*n* = 202), and the *x*-axis is the CoA in the year prior to the recording.

The results of a permutation test clearly showed that signature whistle copying occurred between closely affiliated pairs of animals (*p* = 0.0006). The mean half-weight coefficient of association (CoA; which can range from 0 to 1) for the 10 pairs of animals that copied was 0.8, whereas the mean CoA for non-copiers was 0.4 (figure 2). Interestingly, there were also three instances of copying of whistles that were not signatures between two adult, wild females of low association (see the electronic supplementary material, figure S2). These animals also produced their own signature whistles but no signature whistle copies.

In recordings of four aquarium housed males (forming six possible pairs) at The Seas, one pair also engaged in signature whistle copying. These two individuals showed high levels of synchronous behaviour (23% of 285 min of observation time) in the pool. Synchrony is a sign of social bonding in male bottlenose dolphins [34]. One exchange of signature whistle copying between these males was 30 s in duration: both males emitted the signature whistle of one of them in an interactive sequence consisting of 13 and 11 renditions respectively (see the electronic supplementary material, figure S3). Copying in these individuals was not accompanied by aggressive behaviour (total observation time 16 h with 13 copies produced). The synchrony of the other male pairs was generally lower (7–13% of the observation time). One other pair, however, had a high level of synchrony (26%) but did not engage in whistle copying. Thus, copying does not necessarily occur in bonded males.

### (b) How accurate are vocal copies?

**Figure 3.**
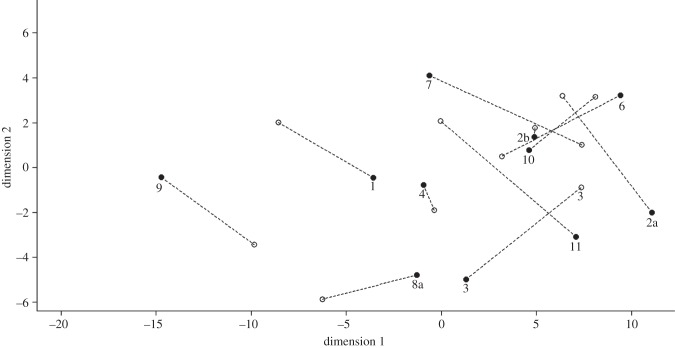
Multi-dimensional scaling plot based on all acoustic parameter measurements. The dotted lines join signature whistle copies (black circles) with the original signature whistles (open circles). Numbers correspond to pairs of animals as given in table 1 (see the electronic supplementary material, table S1). While originals and copies differed significantly in parameters such as start and end frequency as shown here, the overall frequency modulation pattern of the whistle was copied accurately as shown in figure 1.

Frequency parameter measurements of copies produced by 11 animals (one captive and 10 wild animals; two wild copiers were excluded owing to small sample sizes) revealed consistent differences between signature whistle copies and the original, copied signature whistle (table 2 and figure 3). While the overall frequency modulation pattern of the copied whistle showed high similarity to the original (figure 1), copiers introduced consistent variation in single acoustic parameters such as the start or end frequency (see the electronic supplementary material, table S1). In these parameters, copies were often closer to other whistle contours than to the copied signature whistle (figure 3). Individuals varied in the parameters modified; on average 4.4 parameters (range: 1–6) differed significantly between the copy and the original signature whistle. Copies most frequently differed from the original (for 10 of 11 copiers) in mean frequency and maximum frequency (see the electronic supplementary material, table S1). Over half of the copiers also produced copies that differed significantly from the original signature whistle in end frequency (six of 11 copiers) and frequency range (seven of 11 copiers). The copies were equally likely to be higher or lower in frequency than the original. In addition to frequency parameters, one adult male, FB26, altered the number of loops in a multi-looped whistle in his copies of the signature whistle of his alliance partner, adult male FB48. Although FB48 varied his number of loops (range: 3–6), FB26 almost always produced a three-looped copy. The number of loops in FB26's copies and FB48's originals differed significantly (Mann–Whitney: *W* = 152.5, *N*_1_ = 38, *N*_2_ = 35, *p* < 0.0001). All of the signature whistle copies also differed significantly from those of the copiers' own signature whistles in some parameters (mean number of parameters different = 3.54; range: 1–7), whereas other parameters of a copy resembled those of the copier's own signature whistle (mean = 2; range: 0–5).

**Table 2.** Test statistics for all acoustic parameter measurements combined for each copy and original signature whistle comparison. Shown are the sampling statistic of actual combined parameter measurements (observed), and the mean test statistic of combined parameter measurements under the null hypothesis based on 10 000 permutations (expected). Differences between acoustic parameter measurements of vocal copies and original signature whistles are significant at a level of *p* < 0.002.

	observed test statistic	expected test statistic	*p*
Ranier versus copy of Ranier	−7.52	−0.002	0.002
FB48 versus copy of FB48	0.19	−0.007	0.12
FB26 versus copy of FB26	559	0.025	<0.0001
FB20 versus copy of FB20	166	0.43	0.0031
FB122 versus copy of FB122	0.27	0.003	0.1
FB65 versus copy of FB65	1004	0.03	<0.0001
FB55 versus copy of FB55	24 000	0.016	<0.0001
FB35 versus copy of FB35	125	−0.01	<0.0001
FB95 versus copy of FB95	−1439	−0.01	<0.0001
FB155 versus copy of FB155	3 071 589	1.85a	<0.0001
FB177 versus copy of FB177	−2646	−0.0003	<0.0001

### (c) Vocal matching

To further investigate whether copies were emitted in response to the identified model (referred to as the original signature whistle), we investigated whether they were temporally correlated and thus occurred in vocal matching interactions. Vocal matching can be described as a receiver responding to a signal by changing some features of its own vocal behaviour in order to imitate the preceding signal. Bottlenose dolphins had very high vocalization rates during these capture–release events, so it was difficult to judge whether whistles were produced in response to those of other animals. An investigation into the timing of signature whistle copies, however, revealed that the mean time between an original signature whistle and its copy was significantly less than the mean time between an original signature whistle and a copier's own signature whistle (0.94 versus 2.55 s; permutation, *p* < 0.0001). In the long-term captive males, vocal rates were lower, and the matching pattern was clearer: almost all copying events occurred within 1 s after the emission of the original signature whistle by its owner, indicating copies were directed towards the owner of the original signature whistle.

## 4. Discussion

We conducted a large-scale analysis on the occurrence of vocal copying in wild bottlenose dolphins that were briefly caught, sampled and released. This dataset offered a unique opportunity to study the vocal interactions between individuals whose vocal repertoires [14,40] and association patterns had been well documented over decades in the wild [32,42]. In line with previous studies [23,25,26], we found whistle copying to be rare. This is consistent with the idea that signature whistles are used to indicate identity, because such a system would not be sustainable with high copying rates. While a copy could be recognizable as such if it occurred only in specific contexts, aquatic organisms usually have only limited contextual information with the acoustic signals they receive. Frequent copying of signature whistles would therefore render the identity information of the whistle unreliable. The rare copying of signature whistles may, however, be particularly suited to addressing close associates [23–25].

We found that copying occurred primarily in matching interactions between animals with high CoAs outside aggressive contexts, demonstrating that it is an affiliative signal. All pairs of animals that produced signature whistle copies were close associates, with only one pair having a low CoA for the year prior to recording. However, these two males were each other's closest male associate in the 4 year period prior to the recording. Many of the copiers were mother–calf pairs, with both mothers and calves likely to copy one another. While most female calves' signature whistles are distinct from their mothers', males sometimes do sound like their mothers [37]. The signature whistles of the male calves in this study, however, did not resemble those of their mothers (see figure 1 and electronic supplementary material, figure S1). Signature whistles of male alliance partners also tend to become more alike over time [43]. In this study, however, males continued producing their own, non-identical, signature whistles as well as copying the finer details of each other's preferred whistle type. Thus, age, sex and relatedness were not significant factors for the results presented here.

We found no evidence for a deceptive function of signature whistle copies. In animals that are capable of vocal learning, variations can be introduced into a copied signal, allowing encoding of additional information. Bottlenose dolphins produced accurate copies of the frequency modulation pattern of a whistle (figure 1), but introduced fine-scale differences in some acoustic parameters (table 2 and figure 3). As a result, signature whistle copies were clearly recognizable as such. Copies may even carry identity information of the copier, as some individuals maintained some frequency parameters of their own signature whistles in their copies (see the electronic supplementary material, table S1). While these variations may appear subtle, they were generally outside the acoustic variations used by the signature whistle owner itself. Dolphins are clearly capable of detecting such differences in the fundamental frequency as well as the upper harmonics [44,45]. Hence, these copies cannot function in a deceptive manner. Only animals that are familiar with the whistle of the owner would, however, be able to recognize copies. In encounters with unknown animals, a high rate of copying would still lead to confusion, arguing for low rates of copying overall. In fact, wild bottlenose dolphins do not copy signature whistles when encountering other groups of dolphins at sea [46].

Three lines of evidence suggest that active selection may have resulted in the variation found in signature whistle copies. First, bottlenose dolphins are capable of producing almost perfect copies of model sounds [22], suggesting that the variation is not due to limits on copying performance. Second, in experimental copying studies, bottlenose dolphins sometimes alter parameters of copies from one session to the next, and subsequently only produce copies with these novel parameter values [47]. Third, it has been shown that some dolphins introduce novel components such as sidebands to whistle copies, while they are perfectly capable of producing whistles without sidebands at these frequencies [24]. Thus, it is unlikely that variations introduced to copies are merely errors or reflect limitations in copying performance.

A role of vocal learning in the development of signals used in group cohesion and the maintenance of social bonds can be found in a number of social species [3–7,48,49]. The bottlenose dolphin signature whistle stands out in that it is invented by its main producer and can only be shared by animals who had experience with the inventor. Besides humans, bottlenose dolphins appear to be the other main example of affiliative copying with such individually specific learned signals, although some parrot species do use vocal learning to develop labels for social companions [50–52] and therefore deserve further investigation in this context. Further studies are also needed to elucidate whether copying such signals is different from sharing learned contact calls or adjusting acoustic parameters in communal displays as found in other birds and primates. Bottlenose dolphins can be trained to use vocal copies of novel, arbitrary sounds to refer to objects [22]. It is not yet known whether they use learned signals in this way in their own communication system. However, bottlenose dolphins have been found to copy signature whistles of animals that are not present in their group [27]. It is possible that signature whistle copying represents a rare case of referential communication with learned signals in a communication system other than human language [12]. Future studies should look closely at the exact context, flexibility and role of copying in a wider selection of species to assess its significance as a potential stepping stone towards referential communication.
